# Capitalizing on Community Resources to Build Specialized Behavioral Health Services Together with Persons who are Deaf, Deafblind or Hard of Hearing

**DOI:** 10.1007/s10597-015-9940-y

**Published:** 2015-10-27

**Authors:** Kimberly K. Mathos, Robert Q. Pollard

**Affiliations:** University of Pittsburgh Medical Center, Pittsburgh, PA USA; University of Rochester School of Medicine, Rochester, NY USA

**Keywords:** Deaf, Task force, Service expansion, Community inclusion, Community collective impact

## Abstract

There are relatively few counselors, psychologists, psychiatrists, and social workers who specialize in serving people who are Deaf, Deafblind or hard of hearing in the United States. Professionals that serve minority populations are often an insular group. They tend to network most often with fellow professionals who understand the language and cultural needs of their service population. Such specialized behavioral health providers rarely have the opportunity to interface with “mainstream” program planners, funders and administrators. Consequently, new recovery agendas, best practice models and community reintegration ideas are only slowly integrated into the care of persons who are Deaf, Deafblind or hard of hearing. We describe the development and implementation of a task force comprised of “front line” providers, administrators, county government officials, advocates and consumers that has made strides toward effective change in a local behavioral health care system. Methods employed, successes, barriers and other reflections on the task force’s efforts also are described.

Nationwide, there is growing interest in addressing disparities in behavioral health service delivery to minority populations. Agencies charged with raising public awareness about mental health problems such as The National Alliance on Mental Illness (www.nami.org) and Mental Health First Aid (www.MentalHealthFirstAid.org) have begun to tailor their outreach to certain minority populations. In addition, medical schools are teaching students about sociocultural and sociolinguistic issues relevant to linguistic minorities as well as to disability populations early in their training (Barnett et al. 2011; Humphries et al. 2014).

Hearing loss is the sixth most common medical condition in the non-institutionalized US population with a prevalence rate of 9.4 %. (Collins 1997). About one-fifth of these persons have hearing loss of sufficient severity that they cannot hear spoken language even with amplification (Ries 1994). Persons who are deaf bear an increased burden of mental health problems in comparison to the general population (Fellinger et al. 2012).

Based on extrapolated data from the Gallaudet Research Institute (https://research.gallaudet.edu/Demographics/deaf-US.php), in Allegheny county (the Pittsburgh area in Western Pennsylvania) there are estimated to be about 120,000 people who have hearing loss. About 5000 of these persons likely use American Sign Language (ASL) and about 500 are deafblind. Yet in 2005, there were only about 200 deaf or deafblind consumers enrolled in outpatient mental health care with specialized service providers, according to an unpublished summary report compiled by area behavioral health service providers.

The specialist providers in this region are a close-knit group who have committed their careers to serving persons who are Deaf, Deafblind or hard of hearing. They are fluent in ASL and well-versed in Deaf culture. Some are deaf themselves or have family members who are deaf or hard of hearing. However, this group has historically had few ties to behavioral health program planners, community developers and county and state administrators. Therefore this provider group has had limited capacity to bring resources that existed outside their network to the consumers that they served. Service providers also had limited knowledge of funding streams that could be accessed to expand housing resources or community based rehabilitative services that were developing to serve persons with serious mental illness who had normal hearing in this community.

Providers who served the Allegheny County Deaf community nevertheless knew that there were disparities in behavioral health services on many levels. There were limited opportunities for the local Deaf community to learn about mental health-related issues and existing educational and service resources. Access to evaluation or therapy services as well as to mental illness recovery-oriented services such as mobile therapy, case management, group therapies, drug and alcohol services and housing resources was limited (Mathos et al. 2009). The concept of recovery as defined by Substance Abuse and Mental Health Service Administration, wherein individuals improve their health and wellness, live a self-directed life and strive to reach their full potential seemed to be a concept that was not discussed or realized among deaf and hard of hearing consumer groups locally.

In several states (e.g., Alabama, Missouri and others), lawsuits have served as a “call to action,” bringing together advocates, providers, lawyers and program planners to pursue improvements in public mental health services for deaf and deafblind consumers. In Pennsylvania, such a lawsuit was being readied in 2005.

In contrast to lawsuits, a more proactive approach—the formation of task forces—also has been successful. A number of task forces have been formed to address behavioral and/or physical health disparities affecting the Deaf, Deafblind and hard of hearing populations. In 2004 in Rochester, New York, the Finger Lakes Health System Agency convened a Deaf Health Task Force to examine issues related to the health of Rochester’s deaf population. A Medical Accessibility Task Force was formed in 2006 by the Florida Coordinating Council for the Deaf and Hard of Hearing. The Colorado Commission for the Deaf and Hard of Hearing’s Mental Health and Substance Abuse Task Force worked to advance the “Daylight Project” which, among other accomplishments in 2008, yielded a Standards of Behavioral Health Care guide (http://mhcd.org/resource-library/colorado-daylight-partnership). The document addressed a wide range of issues, including administrative procedures, record keeping, communication access and clinical practice when deaf or hard of hearing persons present for treatment.

Noting the success of such task force efforts, one of the local specialist providers in Western Pennsylvania decided to call to the attention of the county behavioral health administrator that significant disparities existed in services for persons who were deaf, deafblind or hard of hearing in that region. The administrator asked the service provider what could be done about this issue. It was decided that the formation of a task force would be a good way to bring together invested providers and community members to work with payers and program planners to identify and address disparities in behavioral health care services (Butterfoss 2007).

## Methods

### Task Force Membership

A local psychiatrist who served the Deaf community and the county’s behavioral health administrator were named as co-chairs of the task force. After the roles of these co-chairs were defined, significant planning went into identifying the preferred characteristics and expertise of the remaining task force membership. We aimed to select a diverse group of individuals who could represent the broad array of needs of a wide array of people with hearing loss. We also needed program planners and payers to help the task force understand and address issues such as grants and other funding mechanisms.

A twenty-member group was eventually formed, comprised of a mix of advocates, community leaders, providers, administrators, sign language interpreters, audiologists, and payers. Some members represented the interest of Deaf persons who use ASL, other members represented the needs of people who were Deafblind and others represented the interest of people who were hard of hearing. The membership was split in regards to hearing status; about half of the group were Deaf persons who were native users of ASL. A secretary was engaged to assist the group’s work. The county administrator agreed to fund accommodation costs (interpreter services and computer-assisted real-time captioning, CART) and refreshments for task force meetings. It was decided that the group would meet every 2 months.

### Task Force Overview

The twenty member group met for the first time in 2005 in the county Human Services Building. A clearly established agenda was established prior to each meeting.

In the first year of the task force, a series of goals was established by consensus vote. These initial goals spanned six topics: resource awareness, information gathering about consumer needs, public outreach and community education, direct service development, workforce development, and mentoring opportunities for young professionals.

Due to the diversity of the membership and the large size of the task force, it became apparent that forming smaller work groups would be a good way to address the defined goals more efficiently. In the small group setting, the passions and unique skills and expertise of group members could be expressed and better utilized. In these workgroups, a problem solving mindset about ways to address specific goal-related topics was fostered. The activities of these work groups were time-limited. Some members participated in several work groups over the course of a year while other members participated only in the large task force meetings. It should be noted, that in the small group setting, hearing and Deaf professionals could learn strategies to better communicate. Despite the use of CART and interpreter services for all meetings, inherent differences in styles of communication and relaying information became evident.

In certain work groups, members from outside the task force were invited to help members better understand or address a particular issue. Work group meetings were often chaired or co-chaired by a person who was deaf, deafblind or hard of hearing. In this manner, work group leaders gained opportunities for leadership skill development and learned how to interface with program administrators. In one work group, for example, a domestic violence counselor chaired the small group, together with a deaf vocational rehabilitation counselor.

Since 2005, a new series of goals has been established each year, after reflection on the goals that were or were not achieved the previous year.

## Outlining Goals and Creating Group Cohesion

In his books, (2003, 2012), Neil Glickman warns about potentially detrimental dynamics of power and control that are inherent in forming work groups comprised of a mix of deaf, hard of hearing and hearing people. Members learned that such things as proper seating arrangement and ground rules for communication were imperative to establish at each meeting. Such things as recognizing the time delay between a hearing person’s comments and the interpretation into ASL were important. Chair persons for the meeting needed to ensure that all comments were fully interpreted and that there was time for feedback before a change in topics occurred.

Early on, it was decided that developing group cohesion in the task force was an important first step in our work together. In 2005, the task force created a membership directory so that members could contact each other as ideas or questions arose between meetings.

As noted, task force members voted on a list of potential goals that the group had brainstormed. Following a procedure adapted by Yoo et al. (2004), each group member was given three small round stickers. Each sticker represented one vote. The various goals under consideration were listed on large poster-paper taped around the walls of the room and group members walked amongst the papers, choosing how to “spend” their three allotted votes. This exercise gave all group members an equal voice in the initial selection of goals and was very empowering to members who had not engaged in community-related work in the past and traditionally had not had a “voice” in program planning efforts.

After the initial votes were tallied, the goals were categorized and prioritized based on the group’s opinions regarding the potential impact or benefit the new services or suggested ideas would have for the community. After the top goals were established, further discussion regarding the feasibility of each goal took place. Thus, the group considered both the impact of achieving each goal as well as the ease or difficulty of achieving that goal, as recommended by Pollard (1995, 2013).

## Resource Awareness/Community Education

The group decided to focus first on goals that did not require a high level of trust between group members or a high level of time commitment. The first goal chosen was to create a local behavioral health service resource directory, listing those services or providers that were known to be accessible to and experienced in serving deaf, deafblind, and/or hard of hearing persons.

The number of listings in the first resource directory was scant but the directory was well developed and widely publicized. It was shared with payers and clinicians and case managers in the region. The directory was made available both in hard copy and electronically so that it could be readily shared on list serves and sent to other professionals who served deaf and hard of hearing people, including vocational counselors, interpreters, audiologists and rehabilitation counselors. The resource directory could also be posted on local Deaf community list serves. The thoughtful approach to the task force’s first goal led to a dramatic increase in the number of deaf and hard of hearing people who sought behavioral health services. One agency reported an increase of 135 consumers enrolled in their services in the 4 years following the publication of the directory.

The resource directory has been updated annually. It now includes the numbers of consumers served by each agency and the type of services each agency offers. Hence, the directory also serves as a community “report card” regarding the pattern of accessibility and service acceptance over time.

After the resource directory goal was accomplished, it was decided that annual conferences should be held to raise awareness about the unique behavioral health needs of persons with hearing loss or deafblindness. The task force partnered with the Pennsylvania Department of Education to conduct the first conference in 2006. The Department of Education donated the training facilities and teleconferencing equipment. The success of this first conference encouraged task force members to think more regionally and to begin to investigate best practice models outside the state. The conference planning workgroup decided to recruit national speakers for the second conference. Successive conferences have been used as forums to educate local providers and administrators about behavioral health service delivery advancements that had been achieved in other states.

## Information Gathering about Unmet Needs

The next task force goal was to gather consumer opinions regarding the existing behavioral health care services in our region. We aimed to also learn what additional services consumers wanted and what type of health-related information or supports would benefit them. We approached a local managed care company and the Allegheny County Office of Behavioral Health for funds to conduct nine consumer focus groups for these purposes.

A flyer describing the focus group effort was distributed to consumers enrolled in area behavioral health care services. Flyers were also given to family members and to local area support group members. Consumers who volunteered for the focus groups were divided into nine categories, based on consumers’ preferred communication modality and age. In this way, we hoped to maximize group homogeneity. There were two groups of deaf consumers who communicated in ASL Two other groups self-identified as hard of hearing. The members of this hard of hearing group preferred using assistive technology for communication, including amplification and CART to dialogue with others in their group. The other five groups were mixed in terms of preferred communication modality because it was felt that other issues were more unifying for these particular groups. For example, one group was comprised of transition-age consumers (18–21 years old) and another group was comprised of consumers who were deafblind.

A number of service needs were identified as common to all of the focus groups (Mathos et al. 2011). These service needs included: self-advocacy training, accessible housing, group therapies, mobile community therapies, community education/outreach presentations, peer support services, case management services, and a need to raise awareness among providers regarding the unique behavioral health needs of persons with hearing loss and deafblindness.

Other service needs identified were more unique to each particular focus group in question. For example, consumers who communicated in ASL wanted to live in supportive housing programs where staff and peers were also deaf and shared their language. Hard of hearing focus group members suggested the need for more cross-training between audiologists, rehabilitations counselors and behavioral health providers. Many members of this group expressed a desire for providers to become more aware of the correlation between hearing loss and stress and anxiety, and for behavioral health providers to learn more about the benefits of assistive technology in ameliorating some behavioral health symptoms. Deafblind consumers advocated for the development of a Support Service Provider program in the region to assist deafblind consumers who aim to live more independently in the community. Deafblind consumers reported that they had extreme difficulty accessing existing office-based services in the region and that office-based therapists seemed unaware of the day to day struggles inherent for a person that has dual sensory loss. They described that they needed help in the community meeting basic needs of independent living.

## Public Outreach and Teaching

Task force consumers and providers alike identified a need for advocacy training and publicizing information about accessible resources and patient rights. One task force work group was charged with creating an “Accommodation Card” which could be used in behavioral health care settings to inform providers about the communication needs of deaf and hard of hearing people and about their rights to communication access in health care settings. Consumers could access a computer template which had a list of possible accommodations commonly utilized in health care settings. The consumer could then select their own preferred accommodations and list how the health care provider could provide or access these accommodations. The consumer could then carry this card in their wallet.

The work group decided that the newly created Accommodation Card needed an “electronic home.” So another task force workgroup created a website for this purpose (www.healthbridges.info). This website has since evolved into a library of resource listings and videos related to social service and behavioral health resources for persons who are deaf, deafblind or hard of hearing and for providers who serve them.

One workgroup tackled another outreach project. One of the hearing task force members is certified as a Mental Health First Aid Trainer. Mental health first aid (www.MentalHealthFirstAid.org) is a nationally standardized training offered to members of the public to help recognize and assist people who manifest signs and symptoms of mental illness or who are in some type of mental health crisis. Several members of the task force were instrumental in advocating for outreach to the Deaf community to make this training accessible, even on a national level. Locally, two deaf behavioral health providers have now been certified as mental health first aid trainers. They have given mental health trainings to interpreter groups, community groups and at a national conference.

It also was decided that local providers could benefit from learning more about the interplay between hearing loss and mental health. Such “awareness” talks have since been given annually at the University of Pittsburgh School of Medicine to residents in training, at area hospitals, police and crisis centers, jails, and social work and mental health training programs.

## Direct Service Development

Direct behavioral health service expansion has also been a goal of the task force. This more time-intensive work has been made possible only after years of collaboration among the task force members on projects related to outreach, advocacy and networking. These service development efforts have required a high degree of interagency collaboration. Some of our service development ideas were readily achieved while others have been much more labor intensive and less successful than we’d prefer.

It was identified early on that we needed a certified peer support person in our community. Peer support services are specialized services provided by current or former consumers of behavioral health services who are trained to offer support and assistance to others in their recovery and community integration process (Pennsylvania Recovery and Resiliency Bulletin, 2014). The consumer who was chosen by the task force to become our peer support specialist received funds from the Office of Vocational Rehabilitation to support the cost of interpreters while receiving peer support training with a group of hearing cohorts.

Next, an expansion of the number of case managers qualified to serve our local special populations was needed. In 2005, there were only two case managers in Western Pennsylvania who had fluency in ASL. The county Office of Behavioral Health and our local insurance provider recently funded two additional ASL-fluent case manager positions. Three of these case managers are Deaf.

Based on feedback from deafblind focus group consumers, a small mobile therapy grant was received from county “reinvestment funds.” In Pennsylvania, behavioral health managed care organizations that are successful in becoming the primary contractor for the HealthChoices Medical Assistance program are allowed to retain capitation revenues and investment income that was not expended during the contract years which can then be reinvested in programs and services in the local community. Mobile therapy services for deaf, deafblind and hard of hearing consumers were funded for 1 year by these reinvestment funds. Mobile therapy is a type of therapy where services are provided in the community or in the home. This form of therapy makes it much easier for the consumer and the therapist to meet regularly. Transportation barriers to receiving services in office-based settings have been momentous for certain populations, including seniors and deafblind consumers, given the demands of medical transport schedules. The mobile therapy program is now self-sustaining on a fee-for-service basis for consumers who receive medical assistance.

The most labor-intensive task force goal has been the development of supportive housing opportunities for our local population with communication and behavioral health challenges. One member of our task force had expertise in housing development initiatives. He was the natural choice to head the supportive housing workgroup. The first objective of this workgroup was to develop a housing facility to which deaf patients being treated at a regional state mental hospital could be discharged. A community site was chosen which was close to shopping and bus lines. The facility had a small number of units which were made accessible to deaf consumers with serious mental illness. Each of the units has been equipped with doorbell lights, vibrating smoke alarms and videophones.

Other hearing consumers of behavioral health services were housed in this sprawling apartment complex as well. Considerable time and effort was required to recruit ASL-fluent staff to work at this facility. Reinvestment dollars were again primarily used for this initiative.

The following year, the housing workgroup embarked on a second project designed for behavioral health consumers who could live independently in the community. The workgroup leader started a dialogue with Pittsburgh-based Action Housing. The group received low income housing tax credits from the Pennsylvania Housing Finance Agency for the start of their work. Also, together with task force members, the housing leaders acquired a federal grant to complete an eight-unit facility for individuals who could live independently in the community with minimal support. The grant was very competitive but the funders were impressed by the number of community partners collaborating on the project. The apartment unit opened in August, 2013. The building now houses eight Deaf consumers who are in recovery and are able to live independently in the community. Deaf case managers assist these consumers in daily living skills and help them to access appropriate social services.

## Mentoring Tracts and Workforce Development

Task force members have appreciated the need to hire more deaf or ASL-fluent staff to meet the needs of a growing number of consumers in our region who are enrolled in behavioral health services. Therefore, a more recent goal of the task force is workforce development.

A focus group was conducted with professors from the University of Pittsburgh and Gallaudet University. Professors in Linguistics (specifically ASL), interpreter training and counseling field placement advisors shared ideas about potential guest lecturing and shared mentoring tracts that they hoped would attract young students into the field of behavioral health. Outreach lectures have been presented to deaf students in social work programs at Gallaudet and area universities and to area students in ASL courses regarding possible careers in behavioral health and related fields. Mentoring opportunities for students in the fields of medicine, social work, interpreting and rehabilitation counseling are also being developed so that students can become comfortable working with deaf, deafblind and hard of hearing persons with mental illness and intellectual disabilities early in their training. A group of seasoned professionals from the task force which include a psychiatrist, social worker, rehabilitation counselor, audiologist and interpreter frequently provide mentorship to these students. Grants are being sought to formalize this process.

### Reflections on Our Achievements

Butterfoss (2007) writes about the progression of intimacy as task force members begin to work together. Early in the evolution of the task force, easily achievable goals that were universally valued were purposefully emphasized. As small successes were achieved, a focus on interagency and intercultural collaboration was encouraged. Kania and Kramer (2011) describe how social change can sometimes best be achieved when the individual agendas of organizations are put aside in favor of a common agenda to improve all parts of a continuum of need. We have witnessed stages of growth, trust and intimacy between members of the task force over time. More recent goals and projects would not have been possible earlier in the life of the task force.

As resource awareness grew, the number of Deaf and hard of hearing consumers enrolled in behavioral health services grew accordingly. So, too, did the number of employment options for deaf and other ASL-fluent persons to provide behavioral health services to the deaf, deafblind, and hard of hearing population. In 2014, a residential provider agency managed by deaf individuals was established in the region.

The task force work groups presented a unique opportunity to promote leadership skills among deaf and hard of hearing service providers. The work groups have also presented an opportunity for people of different linguistic and cultural backgrounds to develop respect and admiration for the experiences of others. Many members were unaware of the historic oppression of the Deaf community. (Lane 1999) When the task force was initiated in 2005, none of the members were skilled grant-writers. Subsequently, grant writing is a skill that several task force members have developed.

Task force members state that their involvement has been a great cultural learning and collaboration opportunity. Deaf and hard of hearing members have learned much about the need for grant-seeking, budget awareness and service development protocols. Administrators and insurers have learned much about working with persons who are deaf, deafblind or hard of hearing and their linguistic, cultural and communication needs in health care settings. All task force members have learned about the need for interdisciplinary collaboration that is inherent to achieve lasting service advancements.

Program planners have reflected on the lessons learned from their experience working with the task force. They have been able to apply some of the lessons learned in their work with our task force to other minority and disability populations.

## Barriers to Our Work

A significant hindrance to our work has been financial support for accommodation costs for interpreters and CART, both of which were necessary for the diverse members of the task force to communicate with one another effectively. The task force meets six times a year. Work groups also generally meet about six times a year. For all these meetings, “platform” interpreters (for ASL users with good distance vision), tactile interpreters (for deafblind task force members) and CART have been provided. The accommodation costs for the large task force meetings have been funded by the Allegheny County Department of Human Services, averaging about six hundred dollars per meeting.

Some task force members have been passionate about issues that are beyond the scope and mission of the task force. It has been necessary to remind members about our mission and current goals at each meeting. A continual barrier has been the tendency of some agency leaders to focus solely on the isolated interventions of their organization rather than to focus on the need for broader community change.

Other barriers were related to attendance at meetings. Attendance at meetings often meant lost income for practicing clinicians.

Transportation and child care costs for community members have also been a challenge to meeting scheduling.

## Future Goals

As we move forward in our work, it is imperative that we find long-term solutions to fund communication accommodations for task force and work group meetings.

Also, as we expand existing behavioral health services for our consumers, the task force seeks to determine how to make Western Pennsylvania attractive to deaf and other ASL-fluent providers who have advanced degrees in counseling, psychology and psychiatry. Options such as shared employment of qualified professionals or joint positions in teaching and service provision are being considered as we aim to regionalize services.

We have recently attracted a residential behavioral health service agency to our region that is managed and operated by Deaf employees. This agency brings unprecedented experience in hiring and managing Deaf staff. Task force members are excited to learn what role the new agency will have in providing recovery-oriented services in our community. We aim to continue to collaborate with other national behavioral health service providers to learn from their experiences in providing services to deaf, deafblind and hard of hearing people.

There are certain consumer groups who continue to have difficulty accessing the services that currently exist. Specifically, those groups include consumers who have intellectual disabilities, addiction-related needs, transition age youth, deafblind consumers and senior citizens.

We believe that our task force has modeled useful strategies for providers and other citizens who seek to engage minority or disability communities in the process of change. It is imperative to dialogue directly with members of communities that are not traditionally served by the mainstream mental health system. Our experiences in bringing program planners together with community members exemplifies how the power of using existing community resources can be a cost-effective and empowering group endeavor.
